# Efficient PCR Amplification Protocol of Nuclear Microsatellites for Exuviae-Derived DNA of Cicada, *Yezoterpnosia nigricosta*

**DOI:** 10.3389/finsc.2021.696886

**Published:** 2021-07-05

**Authors:** Keisuke Yumoto, Takashi Kanbe, Yoko Saito, Shingo Kaneko, Yoshiaki Tsuda

**Affiliations:** ^1^Sugadaira Research Station, Mountain Science Center, University of Tsukuba, Nagano, Japan; ^2^Systematic Entomology, Graduate School of Agriculture, Hokkaido University, Sapporo, Japan; ^3^Department of Ecosystem Studies, Graduate School of Agricultural and Life Sciences, The University of Tokyo, Tokyo, Japan; ^4^Faculty of Symbiotic Systems Science, Fukushima University, Fukushima, Japan

**Keywords:** non-invasive sampling, cicada exuviae, PCR amplification method, population genetics, microsatellites

## Abstract

Although insect exuviae-based genetics is challenging, it can be a valuable method for obtaining reliable DNA resources by non-invasive sampling. This approach is especially effective when the target species is endangered/endemic or when sampling the adult is difficult. One example is cicadas, which during molt leave their exoskeletons on tree trunks, making them easily collectable. While cicada exuviae-derived DNA has previously been employed for mitochondrial DNA sequencing, this study aimed to develop a reliable method for the PCR amplification of nuclear microsatellite loci from cicada exuviae derived DNA for application in molecular ecology, conservation and population genetics. Five different PCR amplification protocols were performed, and the fragment patterns compared with those obtained using DNA extracted from adult individuals. Moreover, the relationship between the freshness of the exuviae and genotyping success was evaluated. *TaKaRa LA Taq* provided the best performance in the PCR amplification of DNA isolated from cicada exuviae and the electropherogram showed a clear fragment pattern that was equivalent to that obtained from the DNA extracted from the adult individual. This result suggests that cicada exuviae-derived DNA can be amplified by PCR and that multiple independent loci of nuclear DNA microsatellite markers can be easily genotyped. This study demonstrates that fresh cicada exuviae provide high quality DNA, which can be used for microsatellite genotyping. The methods developed in this study are applicable not only for cicada but other insect species for which exuviae are available. Thus, this study can make a significant contribution to insect sciences.

## Introduction

The use of samples collected by non-invasive methods has become an increasingly important approach in molecular ecology, phylogeny, conservation and population genetics [e.g., (1, 2)]. These procedures allow us to perform genetic analysis without harming the target species and their populations, which is especially important when genetic analysis is conducted on endangered and/or endemic species, or the sample collection of adult individuals is difficult. In insects, the exuviae are a well-known example of a non-invasive yet reliable source of DNA in various species (1). For example, Keller et al. (3) developed and tested microsatellite markers for an endangered dragonfly by using their exuviae, Lefort et al. (4) employed the exuviae of scarab larvae collected soon after molt to successfully conduct DNA extraction and PCR amplification for species identification, and Inoda et al. (5) demonstrated that the genomic DNA isolated from larval exuviae of diving beetles could be used in mitochondrial DNA (mtDNA) sequencing for species identification.

Cicadas (Hemiptera: Cicadidae) leave their exoskeleton on a place where the final instar nymphs molt, such as tree trunks or leaves. The cicada exuviae can be easily collected by non-invasive sampling methods and they can be used not only to identify the cicada species by morphological characteristics, such as their body size and the antenna, but also to distinguish the sex [e.g., (6, 7)]. In addition, exuviae provide evidence for the distributional range of species because one exuviae indicates that an adult individual has successfully emerged. Therefore, cicada exuviae are mainly known for their usefulness in ecological studies [e.g., (6, 8)]. However, recent studies have started to examine the potential for cicada exuviae to be utilized in genetic studies (1, 9, 10). In these previous studies, genomic DNA was isolated from cicada exuviae and used to PCR amplify a few regions of mtDNA for species identification (9, 10). Nguyen et al. (1) developed an efficient method of DNA extraction from cicada exuviae and highlighted its potential application in various aspects of genetic research. Nevertheless, because of its difficulty, until now there have been no reports of the development of a method to PCR amplify nuclear DNA extracted from cicada exuviae. Mitochondrial DNA has high copy numbers in cells and its mutation rate is higher than that of nuclear DNA (11); thus, mtDNA variation has been examined in many phylogeographic studies (12). However, because mtDNA is maternally inherited and regarded as a single genome without recombination, the obtained genetic information is limited (e.g., the migration of female individuals and the genealogy of a single locus). Therefore, in order to obtain more detailed population genetic information, such as genetic diversity, structure and past demographic history, the examination of nuclear DNA from cicada exuviae is desirable. As nuclear DNA is bi-parentally inherited with recombination, multiple independent loci can be employed to evaluate genetic diversity within/among populations, assess genetic variation between emergence years, and investigate unknown aspects of the life history of cicada species (1).

The aims of this study were: (1) to develop a reliable method for the PCR amplification of nuclear microsatellite loci from cicada exuviae derived DNA; and (2) to demonstrate the use of exuviae as a non-invasive way to conduct population genetic studies on cicada species. It is thought to be difficult to amplify DNA from cicada exuviae, as the DNA extracts typically contain low and fragmented yields. Therefore, it was anticipated that it would be easier to amplify regions in the nuclear DNA with expected PCR product sizes <200–300 bp, smaller than those in the mtDNA study of Nguyen et al. (1). In particular, the focus of this study was on nuclear DNA microsatellite loci because they can be amplified with a small amount DNA, are highly polymorphic, and cost-efficient. As such they are commonly used in molecular ecology as an effective genetic marker (13). In addition, as they are amplified using specific primers developed for the target species (and their closely related species), the risk of amplifying contaminated DNA from other species is reduced compared to when using universal primers.

## Methods

### Sample Collection

**Figure 1:**
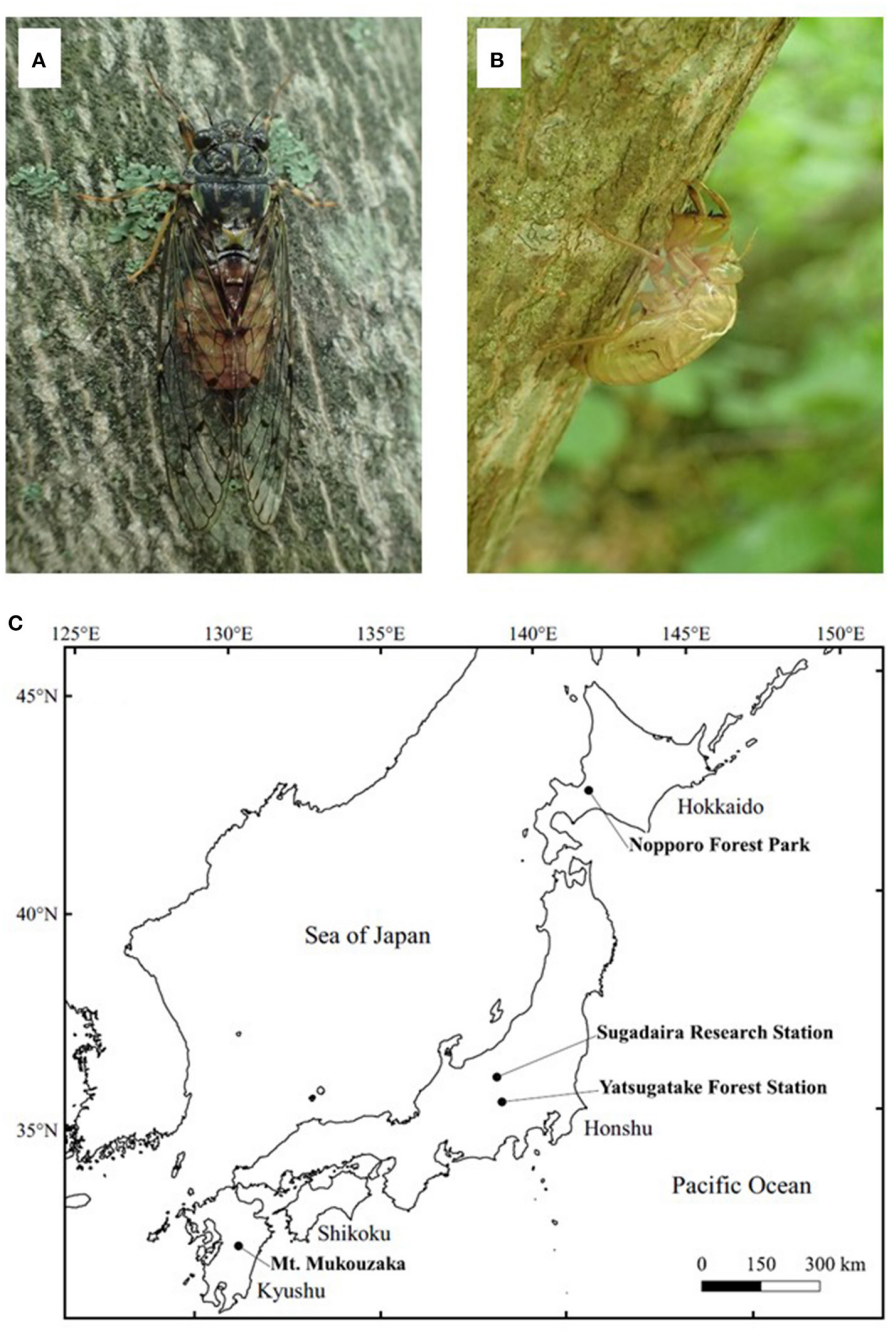
The adult **(A)** and exuviae **(B)** of *Yezoterpnosia nigricosta* and four sampling localities **(C)**. The size of adult and exuviae are about 40 and 20 mm, respectively.

This study used the exuviae of the cicada species, *Yezoterpnosia nigricosta* (Figures 1A,B), which is distributed in subarctic and cool temperate forests in Japan, China and the Russian Far East (14, 15). This species is characterized by its emergence period from spring to early summer (e.g., May–July), much earlier than other cicada species such as *Cryptotympana facialis* and *Graptopsaltria nigrofuscata* (14). A total of 88 samples were collected from four different localities for use in genetic analysis in this study (Figure 1C). Sixty-four fresh exuviae (within a week after emergence) were collected randomly from Sugadaira Research Station, Mountain Science Center (MSC), University of Tsukuba (UT) in central Honshu (36°31' N/138°20' E), Japan from May to July 2018. As sample collection field surveys were conducted every week in this period, the collected exuviae samples were within a week after their emergence. Moreover, eight exuviae were collected randomly from Yatsugatake Forest Station, MSC, UT in central Honshu (35°56' N/138°28' E) in June 2018; eight from Nopporo Forest Park, Hokkaido (43°02' N/141°31' E) and eight from Mt. Mukouzaka, Kyushu (32°34' N/131°06' E) in June and July 2019, respectively. These samples were at most 1- or 2-months post-emergence, assuming the earliest emergence was in the beginning of May. For comparison with the results from the exuviae, four adult individuals were collected from Sugadaira Research Station, MSC, UT in June 2018, two from Yatsugatake Forest Station, MSC, UT in June 2018, two from Nopporo Forest Park in June 2019, and two from Mt. Mukouzaka in July 2019. All dry exuviae and adult samples were kept in individual plastic bags and placed in a cooler box in the field to transport to the laboratory and then, stored at −25°C until DNA extraction.

### DNA Extraction

Total genomic DNA was extracted from the exuviae of 88 individuals using the DNeasy Blood and Tissue Kit (Qiagen, Valencia, CA, USA) according to the manufacturer's instructions, except for the following three modifications. Before starting the DNA extraction procedure, whole exuviae were put into 1.5 mL microcentrifuge tubes together with four 3 mm ceramic beads (YTZ ball, Nikkato corporation) and crushed using a Tissue Lyser 85200 (Qiagen, Valencia, CA, USA) for 30 s. Proteinase K incubation was conducted overnight (15–17 h) and the final elution step was performed twice, adding 25 μL of buffer AE each time in order to obtain a high concentration of DNA.

### PCR Amplification and Microsatellite Genotyping

The microsatellite markers developed for *Y. nigricosta* (15) were used for genotyping. A preliminary PCR amplification test was carried out using the Type-it Microsatellite PCR Kit (Qiagen, Valencia, CA, USA). For multiplex PCR of microsatellite loci, each 5.0 μL reaction contained 2.5 μL of 2 × Type-it Multiplex PCR Master Mix, 0.5 μL of primer mix (0.2 μM of forward primer with fluorescent dye and 0.2 μM of reverse primer), 1.0 μL of RNase-free water and 2–10 ng/μL of genomic DNA. The initial PCR test showed that the fragment patterns of two loci (TeNi002 and TeNi047) were not clear enough for genotyping, probably due to their relatively longer fragment size (228–288 bp compared to 80–180 bp). Since DNA isolated from exuviae is likely to be degraded and fragmented, loci with shorter fragment sizes tend to amplify more easily (16, 17). In order to reduce the fragment size to <200 bp for these two loci, primers for each locus were redesigned using Primer3 version 4.0.0 with default settings (18). Consequently, 17 loci were tested, including the newly developed primers, of which 12 loci with good results were examined in this study (Supplementary Table 1).

Four types of *Taq* with different amplification abilities and five PCR protocols were employed to amplify cicada exuviae-derived DNA as follows (Supplementary Table 2):

1. Type-it Microsatellite PCR Kit (Qiagen, Valencia, CA, USA): A 5.0 μL reaction containing 2.5 μL of 2 × Type-it Multiplex PCR Master Mix, 0.5 μL of primer mix (0.2 μM of forward primer with fluorescent dye and 0.2 μM of reverse primer), 1.0 μL of RNase-free water and 2–10 ng/μL of genomic DNA. The PCR consisted of an initial denaturation at 95°C for 5 min; 40 cycles of denaturation at 95°C for 30 s, annealing at 57°C for 90 s and extension at 72°C for 30 s; and a final extension at 60°C for 30 min.
2. QIAGEN Multiplex PCR Kit (Qiagen, Valencia, CA, USA) Protocol 1: A 5.0 μL reaction containing 2.5 μL of 2 × QIAGEN Multiplex PCR Master Mix, 0.5 μL of primer mix (0.2 μM of forward primer with fluorescent dye and 0.2 μM of reverse primer), 1.0 μL of RNase-free water and 2–10 ng/μL of genomic DNA. The PCR consisted of an initial denaturation at 95°C for 15 min; 40 cycles of denaturation at 94°C for 30 s, annealing at 57°C for 90 s and extension at 72°C for 90 s; and a final extension at 72°C for 10 min.
3. QIAGEN Multiplex PCR Kit Protocol 2: A 5.0 μL reaction containing 2.5 μL of 2 × QIAGEN Multiplex PCR Master Mix, 0.5 μL of primer mix (0.2 μM of forward primer with fluorescent dye and 0.2 μM of reverse primer), 1.0 μL of RNase-free water and 2–10 ng/μL of genomic DNA. The PCR consisted of an initial denaturation at 95°C for 15 min; 40 cycles of denaturation at 94°C for 30 s, annealing at 57°C for 90 s and extension at 72°C for 1 min; and a final extension at 60°C for 30 min.
4. *TaKaRa Ex Taq* (TaKaRa Bio Inc.): A 5.0 μL reaction containing 0.025 μL of *TaKaRa Ex Taq*, 0.5 μL of 10 × *Ex Taq* Buffer, 0.4 μL of dNTP Mixture, 0.5 μL of primer mix (0.2 μM of forward primer with fluorescent dye and 0.2 μM of reverse primer), 2.575 μL of RNase-free water and 2–10 ng/μL of genomic DNA. The PCR consisted of an initial denaturation at 94°C for 1 min; 35 cycles of denaturation at 94°C for 30 s, annealing at 57°C for 1 min and extension at 72°C for 1 min; and a final extension at 72°C for 1 min.
5. *TaKaRa LA Taq* (TaKaRa Bio Inc.): A 5.0 μL reaction containing 0.05 μL of *TaKaRa LA Taq*, 0.5 μL of 10 × LA PCR Buffer II, 0.5 μL of 25 mM MgCl_2_, 0.8 μL of dNTP Mixture, 0.5 μL of primer mix (0.2 μM of forward primer with fluorescent dye and 0.2 μM of reverse primer), 1.65 μL of RNase-free water and 2–10 ng/μL of genomic DNA. The PCR consisted of an initial denaturation at 94°C for 1 min; 30 cycles of denaturation at 94°C for 30 s, annealing at 57°C for 1 min and extension at 72°C for 2 min; and a final extension at 72°C for 3 min.
  For each protocol, eight exuviae collected within a week after their emergence from Sugadaira Research Station were chosen for analysis. Fragment sizes were determined using an ABI PRISM 3130 Genetic Analyzer (Applied Biosystems, Foster City, CA, USA) and GeneMarker software (SoftGenetics, State College, PA, USA) with GeneScan 500 LIZ dye Size Standard v2.0 (Applied Biosystems). To evaluate PCR success, the electropherograms of the exuviae amplified by the five different methods mentioned above were compared with those of the adults amplified by the Type-it Microsatellite PCR Kit. Then, the exuviae-derived DNA of 88 individuals collected from four localities was amplified using the PCR method with the best performance (see results). In order to evaluate the relationship between the freshness of the exuviae and the success rate of the PCR amplification, the PCR success rate of 64 fresh samples (collected within a week after emergence from Sugadaira Research Station) was compared to that of 24 samples of various ages [time since emergence is unknown as sampling was carried out just once during the emergence period at three different localities: Yatsugatake Forest Station, Nopporo Forest Park and Mt. Mukouzaka (eight samples from each site)].

## Results

### Efficient PCR Amplification Method

**Figure 2:**
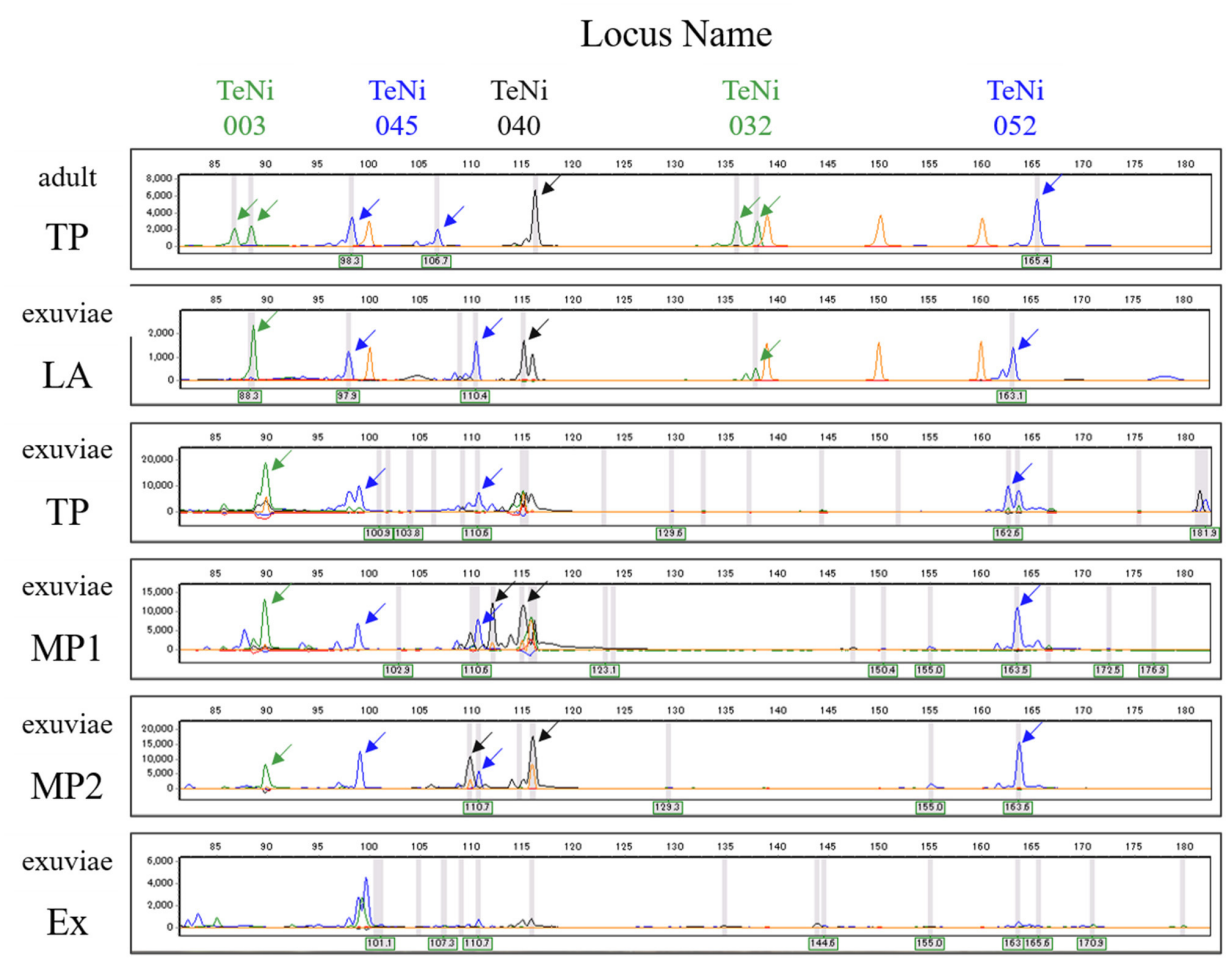
Electropherogram showing amplified alleles at five microsatellite loci (TeNi003, TeNi045, TeNi040, TeNi032, and TeNi052) for DNA extracted from adult (top) and exuviae (the second onwards). Adult and exuviae samples are not the same individual. Electropherograms for each protocol show the results from the same individual. Each gray line represents the peak which the analysis software recognized as an allele. Each peak representing an allele is indicated with an arrow and orange peaks correspond to the size standard. The other annotations represent *TaKaRa LA Taq* (LA), Type-it Microsatellite PCR Kit (TP), QIAGEN Multiplex PCR Kit Protocol 1 (MP1), QIAGEN Multiplex PCR Kit Protocol 2 (MP2), and *TaKaRa Ex Taq* (Ex).

The best performing PCR for the amplification of exuviae-derived DNA of *Y. nigricosta* was the protocol using *TaKaRa LA Taq* (Figure 2). For this protocol the electropherograms (Figure 2) showed clear fragment patterns that were equivalent to the adult-derived DNA. Three of the other protocols: Type-it Microsatellite PCR Kit, and QIAGEN Multiplex PCR Kit Protocols 1 and 2, were also relatively easy to genotype. However, noise fragments, which were not present in the adult-derived DNA, were often detected and interfered with genotyping. The *TaKaRa Ex Taq* protocol performed poorly, with no clear fragment pattern observed. Although the success of PCR amplification was sample dependent in the *TaKaRa LA Taq* protocol, the genomic DNA extracted from cicada exuviae of *Y. nigricosta* was successfully genotyped at all of the 12 microsatellite markers. A total of 69 out of the 88 exuviae collected in this study were successfully amplified for at least one locus, meaning an overall DNA extraction success rate of 78.4% (Table 1). Location specific success rates were 89.1% for Sugadaira Research Station, 25.0% for Nopporo Forest Park, 25.0% for Yatsugatake Forest Station and 100.0% for Mt. Mukouzaka.

**Table 1:** The numbers of successful individuals for DNA extraction and PCR amplification.

**Sampling location**	***N***	**DNA extraction**	**PCR amplification success**		
			**100% (12 loci)**	**75% (9 loci)**	**50% (6 loci)**
Sugadaira Resarch Station	64	57	3	10	20
Yatsugatake Forest Station	8	2	0	0	1
Nopporo Forest Park	8	2	0	0	1
Mt. Mukouzaka	8	8	0	0	3
Total	88	69	3	10	25

*N, number of collected individuals. Samples from Sugadaira Research Station were collected within a week after emergence. At three different localities, time since emergence is unknown as sampling was carried out just once during the emergence period*.

### PCR Success Rate

**Figure 3:**
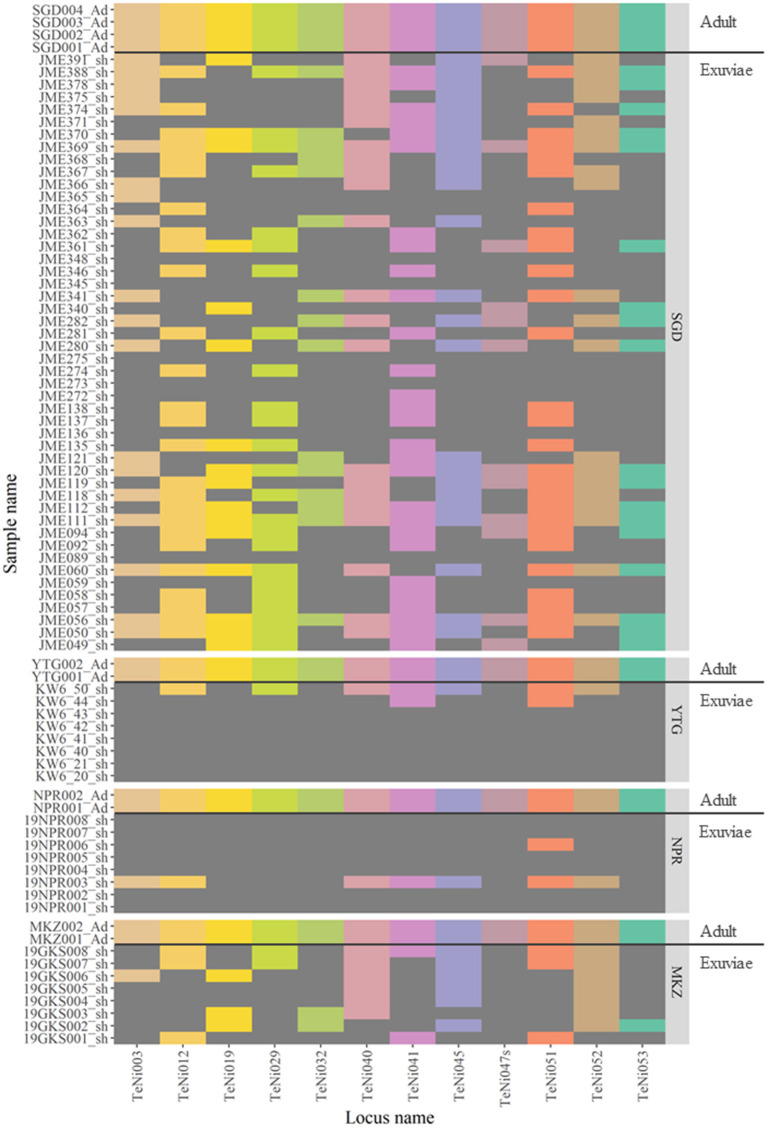
Heatmap showing the genotyping results of 10 adults and 88 exuviae individuals based on 12 microsatellite loci. Colored areas for each locus indicate successful genotyping and dark gray areas where genotyping failed. Population codes are SGD, Sugadaira Research Station; YTG, Yatsugatake Forest Station; NPR, Nopporo Forest Park; MKZ, Mt. Mukouzaka.

The *TaKaRa LA Taq* protocol was used to assess the PCR success rate for 12 nuclear DNA microsatellite markers in 10 adult and 88 exuviae samples from 4 localities (Figure 3). The success of PCR amplification for adults was 100% (12 of 12 loci), however, for exuviae was categorized into three groups: 100% (12 of 12 loci), ≥75% (≥9 of 12 loci), and ≥50% (≥6 of 12 loci) amplification (Table 1). Only three individuals from Sugadaira Research Station showed 100% amplification. Twelve samples from Sugadaira Research Station displayed >75% amplification. The >50% amplification category included 20 samples from Sugadaira Research Station, one from Nopporo Forest Park, one from Yatsugatake Forest Station, and three from Mt. Mukouzaka. Thus, the success rates of the PCR amplification in the three categories (100%, ≥75%, and ≥50%) were 4.7, 15.6, and 31.3%, respectively, in Sugadaira Research Station. The values in Nopporo Forest Park and Yatsugatake Forest Station were 0.0, 0.0, and 12.5%, respectively, and in Mt. Mukouzaka were 0.0, 0.0, and 37.5%, respectively. The PCR success rate was also evaluated for each locus (Supplementary Table 3), and showed that the same loci were successfully amplified among individuals from different sampling locations.

## Discussion

This study demonstrated that genomic DNA can be extracted from cicada exuviae and used to obtain genetic data from multiple independent loci of nuclear DNA microsatellite markers. Although it was confirmed that all five protocols worked well for PCR amplification of adult samples from the four locations and the fragment patterns were basically identical among the protocols (data not shown), the results indicated that the best PCR protocol for microsatellite genotyping of the exuviae-derived DNA was that which used *TaKaRa LA Taq* (Figure 2). Although previous studies have proven that DNA isolated from insect exuviae can be amplified by microsatellite markers in other insect species, such as honey bees (19), damselflies (20), and dragonflies (3, 21), this is the first report in cicada species.

There were several samples for which DNA extraction from the exuviae failed (defined here as unsuccessful amplification across all microsatellite loci, Table 1), suggesting that only low quantities of DNA are left inside cicada exuviae after molt and that the DNA may also contain contaminants that inhibit PCR amplification, such as humic acids in the soil, pointed out by Nguyen et al. (1). In addition, cicada species spend their juvenile period (lasting several years) in the ground and following emergence their exuviae are exposed to a variety of environments, therefore, DNA from other species, such as fungi and bacteria, are often extracted with the target species DNA (1). This problem has also been reported in aquatic insects. Kranzfelder et al. (22) collected chironomid pupal exuviae from water and reported that although the success of DNA extraction and PCR amplification was high, amplification of the target sequence was less successful. Based on these results, the authors concluded that contamination most likely occurs *in situ* at the sampling location. Moreover, OŽana et al. (2) conducted a BLAST analysis of sequences obtained from the exuviae of dragonflies and revealed that these samples contained DNA contaminants from other organisms, such as ciliates, algae, fungi, or bacteria. These previous studies conducted mitochondrial or nuclear DNA sequencing analysis using universal primers, thus non-target DNA is often detected. In order to overcome this contamination issue, the current study employed species-specific (including closely related species) microsatellite primers. Although it is possible the genomic DNA isolated from exuviae in this study contains material from other organisms (21), non-target species DNA would not amplify with *Y. nigricosta*-specific molecular markers. Thus, failure of the PCR suggests that the DNA extraction was not successful or that there was a very small amount of DNA inside cicada exuviae, which was insufficient for PCR [despite the DNA yield (10–40 ng/μL) being determined *via* spectrophotometry (NanoDrop One, Thermo Fisher Scientific)].

To improve the success rate of PCR amplification, freshness of the exuviae samples is important. The results showed that the fresh exuviae of *Y. nigricosta* provided high quality DNA that could be used for microsatellite genotyping (Table 1). This result was supported by Lefort et al. (4) who used the larvae exuviae of scarabs collected immediately after emergence and reported that DNA extraction and PCR amplification was successful in all tested samples. In contrast, Nguyen et al. (1) used cicada exuviae stored at room temperature for 14 months after field collection and could not obtain good results, with only 30.0% sequencing success. Thus, it is important to collect fresh exuviae and also carefully preserve them under dry conditions in a freezer at or below −25°C until DNA extraction, thereby reducing the negative impacts of collection and storage on genomic DNA extraction from exuviae. Similarly, Nguyen et al. (1) and OŽana et al. (2) discussed ways to increase DNA isolation, PCR amplification and genotyping success, recommending the use of fresh or better-preserved samples. In our study, despite DNA being successfully isolated from the cicada exuviae, the success or failure of PCR amplification and genotyping depended on the condition of the samples. Although it is not easy to evaluate the condition of the exuviae by appearance, the condition likely depends on the length of time since emergence and the weather conditions during that period. For example, DNA could be damaged by environmental factors, such as rain and/or sunlight. The higher PCR success rate was achieved for Sugadaira Research Station samples because the exuviae were fresher (less than a week old) than those collected from the other three study sites, where exuviae might have been left in the field under varying environmental conditions for most 1 or 2 months. Moreover, in order to account for the variable success rate, more samples are needed to perform population genetic analyses, which is a practical solution for cicada because it is easy to conduct non-invasive sampling to collect many exuviae.

This study demonstrated that cicada exuviae are reliable genetic resources of nuclear DNA analysis, as suggested in previous mtDNA studies (1, 9, 10). However, as highlighted here and by OŽana et al. (2), the success of PCR amplification depends on the freshness of the samples and the quality of DNA, as discussed above. To overcome this issue, it is recommended to collect and examine more samples collected and stored under varying conditions in order to improve PCR protocols for cicada exuviae-derived DNA. Even so, there is no doubt that cicada exuviae are useful for genetic analysis and genetic information can be obtained without a large environmental impact by utilizing non-invasive sampling methods. Further improving PCR methods for cicada exuviae-derived DNA will be beneficial for various research purposes, for instance, evaluation of genetic diversity within/among populations and estimation of kinship between two individuals. The methods developed in this study are applicable not only for cicada, but also other insect species for which exuviae are available, and thus contributes to the broader research community interested in molecular ecology, conservation and population genetics of insect species.

## Data Availability Statement

The datasets presented in this study can be found in online repositories. The names of the repository/repositories and accession number(s) can be found in the article/Supplementary Material.

## Author Contributions

KY and YT designed the experiment, collected the exuviae samples, and wrote the manuscript, with the contributions from TK, YS, and SK. TK provided the information on how to perform the experiment. KY, YS, and SK performed the experiments. All authors read and approved the manuscript.

## Conflict of Interest

The authors declare that the research was conducted in the absence of any commercial or financial relationships that could be construed as a potential conflict of interest.
